# Effects of peak time of myocardial injury biomarkers on mid-term outcomes of patients undergoing OPCABG

**DOI:** 10.1186/s12872-021-02006-5

**Published:** 2021-04-24

**Authors:** Bo Hu, Fei Gao, Mengwei Lv, Ban Liu, Yu Shi, Xi Chen, Yipeng Feng, Xiaoqi Meng, Zhi Li, Yangyang Zhang

**Affiliations:** 1 Department of Cardiology, Shanghai East Hospital, School of Medicine,Tongji University, Shanghai, China; 2 Cardiovascular Department, Huaiyin Hospital of Huai’an City, Huai’an, China; 3 Shanghai East Hospital of Clinical Medical College, Nanjing Medical University, Shanghai, China; 4 Department of Cardiovascular Surgery, Shanghai East Hospital, School of Medicine,Tongji University, 150 Jimo Road, Shanghai, 200120 China; 5 Department of Cardiology, Shanghai Tenth People’s Hospital, School of Medicine,Tongji University, Shanghai, China; 6 The First Clinical Medical College of Nanjing Medical University, Nanjing, China; 7 The Second Clinical Medical College of Nanjing Medical University, Nanjing, China; 8 Department of Cardiovascular Surgery, Jiangsu Province Hospital, The First Affiliated Hospital of Nanjing Medical University, 300 Guangzhou Road, Nanjing, 210029 China

**Keywords:** Myocardial injury biomarker, Coronary artery bypass grafting, Off pump, Mid-term outcome

## Abstract

**Background:**

With the development of cardiac surgery techniques, myocardial injury is gradually reduced, but cannot be completely avoided. Myocardial injury biomarkers (MIBs) can quickly and specifically reflect the degree of myocardial injury. Due to various reasons, there is no consensus on the specific values of MIBs in evaluating postoperative prognosis. This retrospective study was aimed to investigate the impact of MIBs on the mid-term prognosis of patients undergoing off-pump coronary artery bypass grafting (OPCABG).

**Methods:**

Totally 564 patients undergoing OPCABG with normal courses were included. Cardiac troponin T (cTnT) and creatine kinase myocardial band (CK-MB) were assessed within 48 h before operation and at 6, 12, 24, 48, 72, 96 and 120 h after operation. Patients were grouped by peak values and peak time courses of MIBs. The profile of MIBs and clinical variables as well as their correlations with mid-term prognosis were analyzed by univariable and multivariable Cox regression models.

**Result:**

Continuous assessment showed that MIBs increased first (12 h after surgery) and then decreased. The peak cTnT and peak CK-MB occurred within 24 h after operation in 76.8% and 67.7% of the patients respectively. No significant correlation was found between CK-MB and mid-term mortality. Delayed cTnT peak (peak cTnT elevated after 24 h after operation) was correlated with lower creatinine clearance rate (69.36 ± 21.67 vs. 82.18 ± 25.17 ml/min/1.73 m^2^), body mass index (24.35 ± 2.58 vs. 25.27 ± 3.26 kg/m^2^), less arterial grafts (1.24 ± 0.77 vs. 1.45 ± 0.86), higher EuroSCORE II (2.22 ± 1.12 vs.1.72 ± 0.91) and mid-term mortality (26.5 vs.7.9%). Age (HR: 1.067, CI: 1.006–1.133), left ventricular ejection fraction (HR: 0.950, CI: 0.910–0.993), New York Heart Association score (HR: 1.839, CI: 1.159–2.917), total venous grafting (HR: 2.833, CI: 1.054–7.614) and cTnT peak occurrence within 24 h (HR: 0.362, CI: 0.196–0.668) were independent predictors of mid-term mortality.

**Conclusion:**

cTnT is a better indicator than CK-MB. The peak value and peak occurrence of cTnT are related to mid-term mortality in patients undergoing OPCABG, and the peak phases have stronger predictive ability. *Trial registration*: Chinese Clinical Trial Registry, ChiCTR2000033850. Registered 14 June 2020, http://www.chictr.org.cn/edit.aspx?pid=55162&htm=4.

## Background

Myocardial injury after cardiac surgery, especially coronary artery bypass grafting (CABG), is inevitable. With the improvement in cardiac surgery techniques, off-pump CABG (OPCABG) has become a favorite choice for surgeons in China [1]. This kind of operation can reduce perioperative myocardial injury, but cannot completely eliminate its incidence. Fortunately, most myocardial injuries are asymptomatic and transient accompanied by the increase of cardiac troponin T (cTnT) and creatine kinase myocardial band (CK-MB), and only 1–6.4% of patients are diagnosed with postoperative myocardial infarction [2–5].

Serious myocardial injury is a sensible risk factor for short- and long- term outcomes. CABG is a surgical method of myocardial revascularization, and many factors other than surgical injury can cause myocardial injury, such as reperfusion injury, insufficient myocardial protection, and ischemia. Most studies focus on the relationship between cut-off values of myocardial injury biomarkers (MIBs) and short- and long-term postoperative outcomes [2–7]. Because of the differences in both assessment approaches and patient situations, results of MIBs cut-off values are inconsistent and lack any unified conclusion. Moreover, in the previous studies, MIBs assessments were mostly limited once or twice at a certain time point, and lacked consequent and dynamic observation. The aim of this study was to describe the release profile of perioperative MIBs (including cTnT and CK-MB) and to investigate effects of these changes on the mid-term prognosis of patients undergoing OPCABG.

## Patients and methods

### Patients

**Fig. 1:**
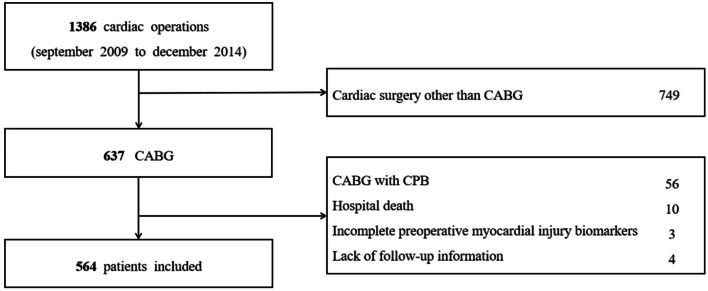
Flowchart of patients’ election

This was a single-centre, observational, retrospective cohort clinical study. Between September 2009 and December 2014, 1386 consecutive patients underwent cardiac operation in the department of cardiovascular surgery. The inclusion criteria were as follows: 1. firstly isolated CABG; 2. no cardiopulmonary bypass (CPB); 3.over 18 years old. The exclusion criteria were as follows: 1. cardiac surgery other than CABG; 2. CABG combined with other procedures; 3. postoperative death; 4. incomplete preoperative MIBs; 5. lack of postoperative follow-up information (Fig. 1).

Finally 564 patients with normal postoperative courses were enrolled. All patients were operated by the same group of surgeons and received routine treatment and nursing care after operation. All patients signed written informed consents for surgical procedures and participation in this study. The study protocols were approved by the ethics committees of Shanghai East Hospital (ID2019067) with the clinical trial registration number ChiCTR-RRC-19014055.

### Cardiac injury biomarker assay

Blood samples for MIB assay were obtained from venous punctures within 48 h before operation and at 6, 12, 24, 48, 72, 96 and 120 h postoperatively. Preoperative MIBs were recorded as baseline values. If a patient recovered well and was discharged within 5 days, MIBs were measured before discharge. MIBs were collected at least until there was a downtrend. Peak values were defined as the highest values measured during postoperative hospitalization. All blood samples were routinely assessed by the same method in the central laboratory of the hospital. The detection limits of cTnT were 0.1–2.0 mg/l and the normal rang was < 0.1 mg/l. The detection limits of CK-MB were 0-400 mg/l, and the normal range was 0-40 mg/l.

### Follow-up

Patients were followed up in the outpatient clinic or by telephone consultation. The first follow-up was one month after discharge, and the second time was at an interval of 2 months. After that, regular follow-up was conducted every 6 months. All cause mortality was recorded and the median (interquartile range) follow-up time was 76.7 (1.0–126.7) months.

### Statistical analysis

Continuous variables were expressed using mean ± standard deviation (SD) if they conformed to normal distribution or were expressed using median with interquartile range (IQR). Categorical variables were summarized as absolute numbers and percentages. Statistical comparisons were performed using t-test, Mann–Whitney U tests for continuous variables, and using Fisher’s exact or Chi-square tests for categorical variables.

Mid-term mortality was calculated and plotted according to the Kaplan–Meier survival curves. All variables were tested by univariate Cox analysis. Variables with *P* < *0.08* were entered into the multivariate analysis. Multivariate Cox regression analysis by using variables from univariate Cox analysis was established to identify the independent risk factors associated with mid-term mortality. Hazard ratios (HR) were expressed with 95% confidence intervals (CI).

Statistical analysis was performed on SPSS 22.0 for windows (IBM, Chicago, USA).Two-tailed *P* < *0.05* was considered significant for all analysis.

## Results

Blood samples were assessed a specific value of MIBs at each monitoring point. The 564 patients had 4163 monitoring points (Some patients recovered well and were discharged within 5 days after operation), and 3527 monitoring points were completed (3527/4163, 84.7%). About 557 (557/564, 98.8%) patients had measurements of MIBs at more than 3 postoperative time points. There were 443 males (78.5%) and 121 females (21.5%) who had normal postoperative courses, with a mean age of 65.3 ± 8.2 years. Moreover, 60 patients died during the 1.0 to 126.7 (median of 75.7) months of follow-up (Table 1).

**Table 1:** Clinical characteristics of entire study patients

Variables	Total (n = 564)
Male sex (n, %)	443 (78.5)
Age (y)	65.30 ± 8.20
Weight (kg)	70.05 ± 10.39
Height (cm)	166.81 ± 6.77
BMI (kg/m^2^)	25.13 ± 3.18
BSA (m^2^)	1.76 ± 0.16
Types of CAD	
Unstable angina pectoris (n, %)	332 (58.9)
Acute myocardial infarction (n, %)	75 (13.3)
Stable angina pectoris (n, %)	157 (27.8)
Diabetes mellitus (n, %)	201 (35.6)
Hypertension (n, %)	413 (73.2)
Cerebrovascular disease	
Stroke (n, %)	22 (3.9)
Lacunar infarction (n, %)	141 (25.0)
Peripheral vascular disease (n, %)	13 (2.3)
Preoperative atrial fibrillation (n, %)	15 (2.7)
Pulmonary hypertension (n, %)	12 (2.1)
Serum creatinine (μmol/l)	82.51 ± 24.57
Ccr (mL/min/1.73 m^2^)	80.30 ± 25.09
Renal failure (n, %)	4 (0.7)
LVEF (%)	
LVEF ≥ 45% (n, %)	549 (97.3)
LVEF < 45% (n, %)	15 (2.7)
Triple vessel disease (n, %)	505 (89.5)
COPD (n, %)	9 (1.6)
Previous PCI (n, %)	2 (0.4)
NYHA	
I (n, %)	30 (5.3)
II (n, %)	415 (73.6)
III (n, %)	113 (20.0)
IV (n, %)	6 (1.1)
EuroSCORE II	1.79 ± 0.96
Operating time (min)	267.96 ± 64.04
Number of bypass grafts (n)	3.70 ± 1.08
Number of arterial grafts (n)	1.42 ± 0.85
Number of venous grafts (n)	2.28 ± 1.12
Total arterial grafting (n, %)	42 (7.4)
Total venous grafting (n, %)	35 (6.2)
Peak of CK-MB (mg/L)	21.45 (15.00, 32.00)
Peak of cTnT (mg/L)	0.15 (0.10, 0.34)
CK-MB peak occurrence within 24 h (n, %)	382 (67.7)
CK-MB peak value > 32.00 mg/L (n, %)	139 (24.6)
Follow-up time (month)	75.65 (59.88, 93.58)
Mid-term mortality (n, %)	60 (10.6)

*BMI* body mass index, *BSA* body surface area, *CAD* coronary artery disease, *Ccr* creatinine clearance rate, *LVEF* left ventricular ejection fraction, *COPD* chronic obstructive pulmonary disease, *PCI* percutaneous coronary intervention, *NYHA* New York Heart Association, *EuroSCORE II* European system for cardiac operative risk evaluation II, *CK-MB* creatine kinase myocardial band, *cTnT* cardiac troponin T

### MIBs release

**Fig. 2:**
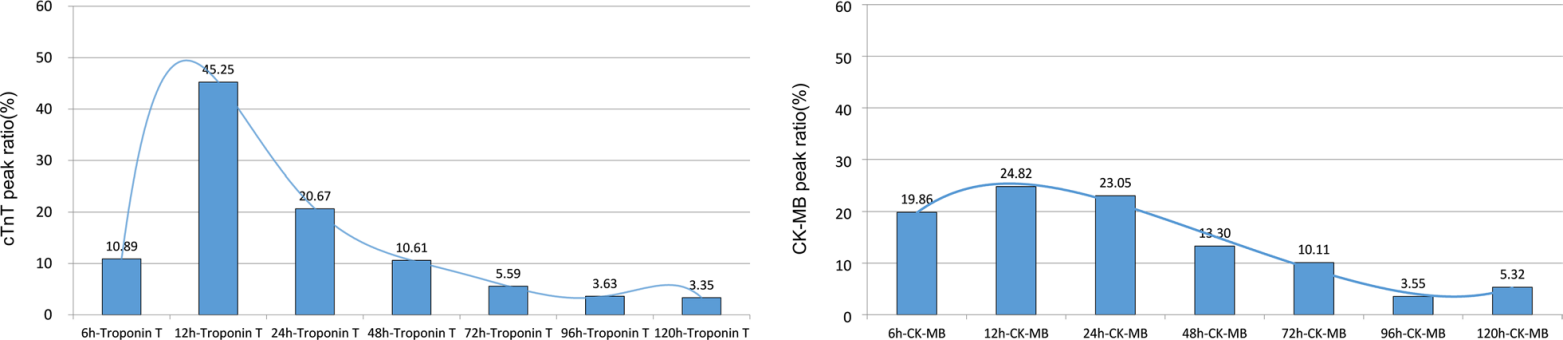
**a** Proportion of cTnT peak at each time point after operation. **b** Proportion of CK-MB peak at each time point after operation

cTnT and CK-MB were measured at 7 monitoring points postoperatively. The release profile of postoperative MIBs increased first and then decreased. The increase of cTnT was more obvious than that of CK-MB. The median peak values of cTnT and CK-MB were 0.15 (IQR: 0.10, 0.34) mg/l and 21.45 (IQR: 15.00, 32.00) mg/l respectively. cTnT peaked within 24 h after operation in 76.8% of the total cohort (Fig. 2a).Similarly, CK-MB peaked in 67.7% of patients within 24 h after surgery (Fig. 2b).

### Univariate and multivariate Cox regression analysis

**Fig. 3:**
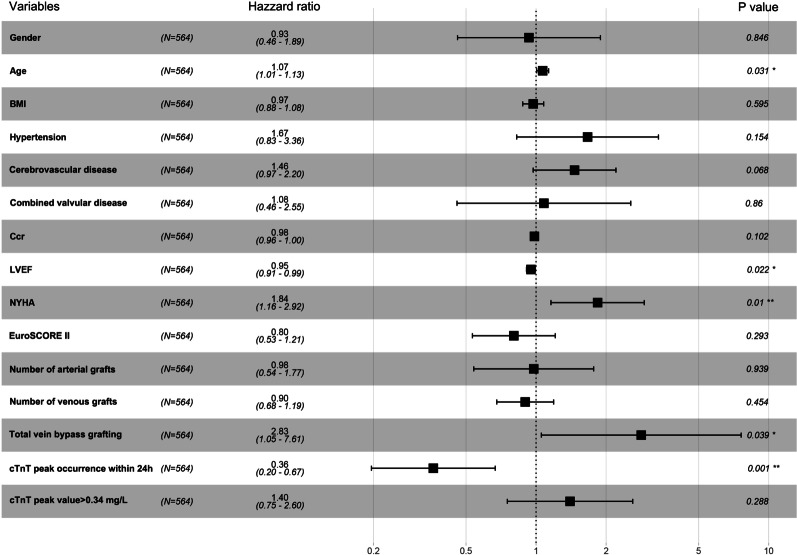
Forest plot of the variables for mid-term mortality

All clinical variables were entered into univariate analysis. Age, cerebrovascular disease, combined valvular disease, preoperative creatinine clearance rate (Ccr), left ventricular ejection fraction (LVEF), New York Heart Association (NHYA), EuroSCORE II, number of arterial grafts, number of venous grafts, total vein bypass grafting, cTnT peak occurrence within 24 h and cTnT peak value > 0.34 mg/l were significantly associated with mid-term mortality. In multivariate Cox regression analysis, age, LVEF, NYHA, total venous grafting and cTnT peak occurrence within 24 h remained significant, and cTnT peak occurrence within 24 h was the strongest predictor (Table 2) (Fig. 3). Interestingly, in multivariate analysis with the same variables but without cTnT peak occurrence within 24 h, cTnT peak value > 0.34 mg/l was a significant independent risk factor (*P* = *0.015*). In univariate Cox analysis, CK-MB-related variables had no significant effect on mid-term mortality (Table 2).

**Table 2:** Univariate and multivariate cox regressions analysis for entire study patients

	Univariate Analysis		Multivariate Analysis excluding (cTnT peak value > 0.34 mg/l)		Multivariate Analysis excluding (cTnT peak occurrence within 24 h)		Multivariate Analysis (Total)	
	HR (95% CI)	*P* value	HR (95% CI)	*P* value	HR (95% CI)	*P* value	HR (95% CI)	*P* value
Sex (n, %)	1.043 (0.564–1.928)	0.894	0.922 (0.455–1.868)	0.822	1.096 (0.550–2.185)	0.795	0.933 (0.460–1.892)	0.847
Age (y)	1.097 (1.058–1.138)	< 0.001	1.060 (1.000–1.122)	0.048	1.076 (1.014–1.141)	0.016	1.067 (1.006–1.133)	0.031
BMI (kg/m^2^)	0.925 (0.850–1.007)	0.071	0.976 (0.880–1.082)	0.976	0.979 (0.883–1.084)	0.681	0.972 (0.876–1.079)	0.595
BSA (m^2^)	0.350 (0.069–1.775)	0.205						
Types of CAD (n, %)	0.717 (0.473–1.088)	0.118						
Diabetes mellitus (n, %)	1.392 (0.830–2.333)	0.210						
Hypertension (n, %)	1.792 (0.927–3.462)	0.083	1.719 (0.855–3.455)	0.128	1.732 (0.864–3.473)	0.121	1.665 (0.826–3.358)	0.154
Cerebrovascular disease (n, %)	1.815 (1.251–2.635)	0.002	1.438 (0.952–2.172)	0.084	1.510 (1.005–2.268)	0.047	1.464 (0.972–2.205)	0.068
Peripheral vascular disease (n, %)	0.889 (0.123–6.433)	0.907						
Combined valvular disease (n, %)	2.385 (1.169–4.864)	0.017	1.057 (0.453–2.466)	0.897	1.073 (0.456–2.525)	0.871	1.081 (0.457–2.553)	0.872
Preoperative atrial fibrillation (n, %)	1.648 (0.401–6.765)	0.488						
Pulmonary hypertension (n, %)	3.130 (0.756–12.951)	0.115						
Ccr (ml/min/1.73 m^2^)	0.968 (0.955–0.981)	< 0.001	0.982 (0.963–1.002)	0.076	0.982 (0.963–1.000)	0.053	0.984 (0.965–1.003)	0.102
LVEF (%)	0.951 (0.918–9.984)	0.004	0.951 (0.911–0.993)	0.023	0.951 (0.909–0.994)	0.028	0.950 (0.910–0.993)	0.022
Number of diseased vessels	0.746 (0.458–1.215)	0.239						
COPD (n, %)	1.133 (0.157–8.187)	0.901						
Previous PCI (n, %)	0.050 (0.000–1.25*10^9)	0.806						
NYHA (n, %)	2.160 (1.403–3.326)	< 0.001	1.781 (1.125–2.821)	0.014	1.903 (1.199–3.019)	0.006	1.839 (1.159–2.917)	0.010
EuroSCORE II	1.695 (1.383–2.078)	< 0.001	0.816 (0.544–1.225)	0.327	0.767 (0.508–1.156)	0.204	0.802 (0.533–1.209)	0.292
Operating time (min)	0.999 (0.995–1.003)	0.486						
Number of bypass grafts	0.928 (0.723–1.191)	0.558						
Number of arterial grafts	0.388 (0.239–0.628)	< 0.001	0.964 (0.537–1.732)	0.903	1.053 (0.582–1.906)	0.865	0.977 (0.540–1.769)	0.939
Number of venous grafts	1.311 (1.037–1.659)	0.024	0.901 (0.680–1.193)	0.465	0.972 (0.734–1.286)	0.841	0.898 (0.678–1.190)	0.454
Total artery bypass grafting (n, %)	0.480 (0.150–1.538)	0.217						
Total vein bypass grafting (n, %)	6.247 (3.077–12.683)	< 0.001	2.855 (1.066–7.647)	0.037	3.036 (1.133–8.135)	0.027	2.833 (1.054–7.614)	0.039
CK-MB peak (mg/L)	1.001 (0.995–1.008)	0.670						
CK-MB peak occurrence within 24 h (n, %)	0.773 (0.461–1.296)	0.328						
CK-MB peak value > 32.00 mg/l (n, %)	1.452 (0.842–2.505)	0.180						
cTnT peak (mg/l)	1.544 (0.936–2.546)	0.089						
cTnT peak occurrence within 24 h (n, %)	0.243 (0.143–0.413)	< 0.001	0.318 (0.181–0.557)	< 0.001			0.362 (0.196–0.668)	0.001
cTnT peak value > 0.34 mg/L (n, %)	1.819 (1.073–3.084)	0.026			2.006 (1.145–3.513)	0.015	1.400 (0.753–2.604)	0.288

*BMI* body mass index, *BSA* body surface area, *CAD* coronary artery disease, *Ccr* creatinine clearance rate, *LVEF* left ventricular ejection fraction, *COPD* chronic obstructive pulmonary disease, *PCI* percutaneous coronary intervention, *NYHA* New York Heart Association, *EuroSCORE II* European system for cardiac operative risk evaluation II, *CK-MB* creatine kinase myocardial band, *cTnT* cardiac troponin T

### Group classification

Since CK-MB related variables had no correlation with the end-point events, cTnT related variables had become the research focus. For further evaluation, patients were divided into four groups according to two criteria (peak values and peak time courses of cTnT):

Group I: cTnT peak occurrence within 24 h, n = 481;

Group II: cTnT peak occurrence after 24 h, n = 83;

Group III: cTnT peak value ≤ 0.34 mg/l (75% percentile), n = 424;

Group IV: cTnT peak value > 0.34 mg/l (75% percentile), n = 140.

### Perioperative variables

Patients in group I showed younger age, larger body mass index (BMI), lower serum creatinine, higher Ccr, lower EuroSCORE II, more arterial grafts, less venous grafts, lower CK-MB and cTnT peak values, lower prevalence of CK-MB peak value > 32.00 mg/l, longer follow-up time and lower mid-term mortality compared with group II (Table 3).

**Table 3:** Clinical characteristics of patients with cTnT peak occurrence within 24 h after operation vs. cTnT peak occurrence more than 24 h after operation

Variables	Group I (n = 481)	Group II (n = 83)	P
Male sex (n*,* %)	380 (79.0)	63 (75.9)	0.525
Age (y)	64.77 ± 8.22	68.41 ± 7.39	< 0.001
Weight (kg)	7.035 ± 10.53	68.30 ± 9.39	0.097
Height (cm)	166.73 ± 6.70	167.29 ± 7.13	0.487
BMI (kg/m^2^)	25.27 ± 3.26	24.35 ± 2.58	0.005
BSA (m^2^)	1.76 ± 0.16	1.74 ± 0.15	0.231
Types of CAD			0.643
Unstable angina pectoris (n*,* %)	284 (59.0)	48 (57.8)	
Acute myocardial infarction (n*,* %)	66 (13.7)	9 (10.8)	
Stable angina pectoris (n*,* %)	131 (27.2)	26 (31.3)	
Diabetes mellitus (n*,* %)	164 (34.1)	37 (44.6)	0.066
Hypertension (n*,* %)	346 (71.9)	67 (80.7)	0.095
Cerebrovascular disease			0.301
Stroke (n*,* %)	18 (3.7)	4 (4.8)	
Lacunar infarction (n*,* %)	115 (23.9)	26 (31.3)	
Peripheral vascular disease (n*,* %)	10 (2.1)	3 (3.6)	0.642
Preoperative atrial fibrillation (n*,* %)	14 (2.9)	1 (1.2)	0.601
Pulmonary hypertension (n*,* %)	8 (1.7)	4 (4.8)	0.153
Serum creatinine (μmol/l)	80.93 ± 20.31	91.66 ± 40.35	0.020
Ccr (ml/min/1.73 m^2^)	82.18 ± 25.17	69.36 ± 21.67	< 0.001
Renal failure (n*,* %)	2 (0.4)	2 (2.4)	0.197
LVEF (%)			0.878
LVEF ≥ 45% (n*,* %)	468 (97.3)	81 (97.6)	
LVEF < 45% (n*,* %)	13 (2.7)	2 (2.4)	
Triple vessel disease (n*,* %)	426 (88.6)	79 (95.2)	0.069
COPD (n*,* %)	7 (1.5)	2 (2.4)	0.868
Previous PCI (n*,* %)	2 (0.4)	0 (0.0)	1.000
NYHA			0.912
I (n*,* %)	26 (5.4)	4 (4.8)	
II (n*,* %)	356 (74.0)	59 (71.1)	
III (n*,* %)	94 (19.5)	19 (22.9)	
IV (n*,* %)	5 (1.0)	1 (1.2)	
EuroSCORE II	1.72 ± 0.91	2.22 ± 1.12	< 0.001
Operating time (min)	266.19 ± 61.80	278.20 ± 75.37	0.115
Number of bypass grafts (n)	3.67 ± 1.09	3.87 ± 1.02	0.119
Number of arterial grafts (n)	1.45 ± 0.86	1.24 ± 0.77	0.028
Number of venous grafts (n)	2.21 ± 1.12	2.67 ± 1.03	< 0.001
Total arterial grafting (n*,* %)	40 (8.3)	2 (2.4)	0.058
Total venous grafting (n*,* %)	26 (5.4)	9 (10.8)	0.058
Peak of CK-MB (mg/l)	19.70 (14.00, 29.05)	32.90 (21.00, 61.00)	< 0.001
Peak of cTnT (mg/l)	0.13 (0.10, 0.26)	0.43 (0.25, 0.81)	< 0.001
CK-MB peak occurrence within 24 h (n*,* %)	327 (68.0)	55 (66.3)	0.757
CK-MB peak value > 32.00 mg/l (n*,* %)	97 (20.1)	42 (50.6)	< 0.001
Follow-up time (month)	77.10 (61.00, 94.20)	69.10 (34.80, 86.60)	0.001
Mid-term mortality (n*,* %)	38 (7.9)	22 (26.5)	< 0.001

*BMI* body mass index, *BSA* body surface area, *CAD* coronary artery disease, *Ccr* creatinine clearance rate, *LVEF* left ventricular ejection fraction, *COPD* chronic obstructive pulmonary disease, *PCI* percutaneous coronary intervention, *NYHA* New York Heart Association, *EuroSCORE II* European system for cardiac operative risk evaluation, *CK-MB* creatine phosphokinase isoenzyme, *cTnT* cardiac troponin T

Group I: cTnT peak occurrence within 24 h after operation; Group II: cTnT peak occurrence more than 24 h after operation

Patients in group III had lower prevalence of hypertension, total vein bypass grafting, CK-MB peak occurrence within 24 h, CK-MB peak value > 32.00 mg/l and lower serum creatinine, higher Ccr, shorter operation time, less venous grafts, lower CK-MB and cTnT peak values, and lower mid-term mortality compared with group IV (Table 4).

**Table 4:** Clinical characteristics of patients with cTnT peak value ≤ 0.34 mg/l vs. cTnT peak value > 0.34 mg/l

Variables	Group III (n = 424)	Group IV (n = 140)	P
Male sex (n*,* %)	341 (80.4)	102 (72.9)	0.059
Age (y)	65.40 ± 8.20	64.99 ± 8.21	0.608
Weight (kg)	70.33 ± 10.51	69.18 ± 10.02	0.255
Height (cm)	166.92 ± 6.68	166.48 ± 7.03	0.502
BMI (kg/m^2^)	25.21 ± 3.25	24.92 ± 2.96	0.351
BSA (m^2^)	1.77 ± 0.16	1.75 ± 0.16	0.256
Types of CAD			0.127
Unstable angina pectoris (n*,* %)	250 (59.0)	82 (58.6)	
Acute myocardial infarction (n*,* %)	50 (11.8)	25 (17.9)	
Stable angina pectoris (n*,* %)	124 (29.2)	33 (23.6)	
Diabetes mellitus (n*,* %)	155 (36.6)	46 (32..9)	0.428
Hypertension (n*,* %)	302 (71.2)	111 (79.3)	0.062
Cerebrovascular disease			0.525
Stroke (n*,* %)	16 (3.8)	6 (4.3)	
Lacunar infarction (n*,* %)	111 (26.2)	30 (21.4)	
Peripheral vascular disease (n*,* %)	8 (1.9)	5 (3.6)	0.249
Preoperative atrial fibrillation (n*,* %)	12 (2.8)	3 (2.1)	0.661
Pulmonary hypertension (n*,* %)	7 (1.7)	5 (3.6)	0.172
Serum creatinine (μmol/l)	80.83 ± 19.83	87.59 ± 34.83	0.005
Ccr (ml/min/1.73 m^2^)	81.58 ± 24.74	76.39 ± 25.81	0.034
Renal failure (n*,* %)	2 (0.5)	2 (1.4)	0.242
LVEF (%)			0.867
LVEF ≥ 45% (n*,* %)	413 (97.4)	136 (97.1)	
LVEF < 45% (n*,* %)	11 (2.6)	4 (2.9)	
Triple vessel disease (n*,* %)	379 (89.4)	126 (90.0)	0.837
COPD (n*,* %)	7 (1.7)	2 (1.4)	0.856
Previous PCI (n*,* %)	2 (0.5)	0 (0)	0.416
NYHA			0.673
I (n*,* %)	25 (5.9)	5 (3.6)	
II (n*,* %)	308 (72.6)	107 (76.4)	
III (n*,* %)	86 (20.3)	27 (19.3)	
IV (n*,* %)	5 (1.2)	1 (0.7)	
EuroSCORE II	1.75 ± 0.94	1.92 ± 1.02	0.075
Operating time (min)	263.00 ± 59.08	282.96 ± 75.44	0.001
Number of bypass grafts (n)	3.68 ± 1.11	3.76 ± 0.97	0.446
Number of arterial grafts (n)	1.44 ± 0.87	1.35 ± 0.80	0.274
Number of venous grafts (n)	2.22 ± 1.12	2.44 ± 1.09	0.044
Total artery bypass grafting (n*,* %)	36 (8.5)	6 (4.3)	0.100
Total vein bypass grafting (n*,* %)	22 (5.2)	13 (9.3)	0.081
Peak of CK-MB (mg/l)	18.00 (13.50, 25.00)	41.00 (28.30, 69.85)	< 0.001
Peak of cTnT (mg/l)	0.11 (0.10, 0.18)	0.73 (0.44, 1.12)	< 0.001
CK-MB peak occurrencewithin 24 h (n*,* %)	272 (64.2)	114 (78.6)	0.002
CK-MB peak value > 32.00 mg/l (n*,* %)	47 (11.1)	92 (65.7)	< 0.001
Follow-up time (month)	75.75 (60.63, 93.34)	75.45 (46.05, 95.33)	0.578
Mid-term mortality (n*,* %)	37 (8.7)	23 (16.4)	0.010

*cTnT* cardiac troponin T, *BMI* body mass index, *BSA* body surface area, *Cr* creatinine, *Ccr* creatinine clearance rate, *COPD* chronic obstructive pulmonary disease, *PCI* percutaneous coronary intervention, *CAD* coronary artery disease, *NYHA* New York Heart Association, *LVEF* left ventricular ejection fraction, *EuroSCORE II* European system for cardiac operative risk evaluation II

Group III: Peak of postoperative cTnT ≤ 0.34 mg/l; Group IV: Peak of postoperative cTnT > 0.34 mg/l

### Follow-up

All patients were followed up after discharge. The median follow-up time was 76.7 months. The 1-, 5- and 8-year survival rates were 98.0 ± 0.6%, 96.6 ± 0.8% and 86.5 ± 2.0%, respectively. During the follow-up period, 60 patients died, accounting for 10.6% of the total cohort.

**Fig. 4:**
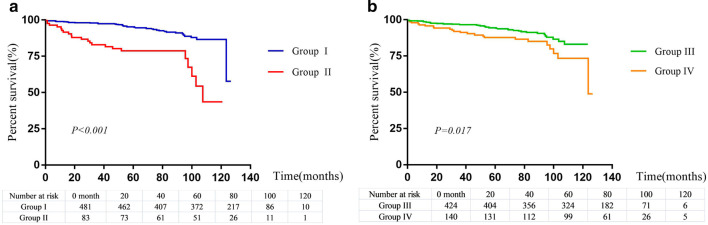
**a** Kaplan–Meier survival curves for mortality in group I and group II. **b** Kaplan–Meier survival curves for mortality in group III and group IV

The Kaplan–Meier analyses showed that group I versus group II (*P* < *0.001*), and group III versus group IV (*P* = *0.017*)had better survival (Fig. 4a, b).

## Discussion

The time course of MIBs increased first and then decreased after OPCABG. The peak value of cTnT in most patients appeared at 24 h after surgery. We analyzed the risk factors of mid-term outcome in 564 patients undergoing OPCABG with normal postoperative courses and started from both the peak values and time courses of MIBs. It was found that postoperative peak values and peak occurrence time of cTnT were significant predictors of mid-term mortality. Through literature search and review, we know this is the first report in China to clearly put forward that the peak occurrence time is more predictive than absolute value of peak value in OPCABG patients.

Myocardial injury is an inevitable and common phenomenon during and after CABG. Available literature on this aspect has clearly reported, but not limited to, the insufficient myocardial protection, surgical procedure injuries, ischemia and factors related to patient states [8–10]. CABG is one of the most common types of cardiac surgery and the number of which is largest in cardiac operation all over the world [11, 12]. Due to the reduction of extracorporeal circulation procedures, OPCABG fundamentally avoids the problem of myocardial protection. The off-pump pattern can reduce postoperative complications and mortality, which is very suitable for the relatively backward cardiac surgery techniques in China. Therefore, OPCABG is very popular in China, and up to 60% of severe CAD patients select OPCABG [1]. It is important and significant to better understand the role of MIBs in mid-term prognosis of patients undergoing OPCABG.

MIBs are the important indicators of postoperative myocardial injury in clinical practice. The cTnT with very high sensitivity and specificity is suitable for early warming of myocardial injury [5, 6, 13, 14]. Most of the current articles focus on the relationship between specific peak values of MIBs and short- or long-term prognosis. Due to differences in assessment approaches, time and number of assessment, there is no unified understanding of this issue. Most literature involves only 2–3 measurements at certain time points after operation, including many types of cardiac surgeries and mixed postoperative death cases [2–5, 7, 15–17]. Those factors make it difficult to understand the profile of MIBs after surgical performances. In this study, to focus the dynamic changes of MIBs after OPCABG, we excluded death cases and observed the MIBs in a continuous manner at 7 consecutive points after operation.

Due to the distinction in many factors such as instruments, testing approaches and testing environment, there could be absolute differences in the test value of MIBs for patients undergoing cardiac surgery. However, multiple clinical studies have shown that the peak occurrence time did not vary with different testing factors. Using the time of peak as a variable, the absolute value difference could be offset. As reported, the peak cTnT occurred between 24 and 48 h after cardiac surgery regardless of different testing factors, which was also confirmed by our findings [4, 5, 9, 18]. In our cohort study, the cTnT peaked at 24 h after OPCABG in most cases (76.82%) in the patients with uneventful courses. The proportion of patients whose CK-MB peaked within 24 h after operation was 67.73%. Patients with maximal peak values had a delayed increase time of peak occurrence, which is similar to another report [15].

Traditional MIBs include cTnT, cTnI and CK-MB. The specificity and sensitivity are higher for both cTnT and cTnI than CK-MB [5, 19, 20]. In this study, although the change trends of CK-MB were similar to those of cTnT, the values and occurrence time of CK-MB peaks were not the risk factors of mid-term mortality (Table 2). This result also reflects that cTnT is superior over CK-MB [18]. The possible explanations may be that cTnT is more specific and sensitive than CK-MB in myocardial injury. The skeletal muscle injury can also cause the increase of CK-MB [21].The univariate Cox regression model suggested the value and occurrence time of cTnT peak were both risk factors of mid-term mortality. The results of the current study suggest that several factors influence the mid-term prognosis of OPCABG. Age, LVEF, NYHA, total vein bypass grafting and cTnT peak occurrence within 24 h clearly influence the mid-term mortality. As for the effects of MIBs on the long-term prognosis of CABG patients, Camilla et al. [7] also found the cTnT value at 44 h postoperatively was more predictive than the values at 7 or 20 h or the peak values. Unfortunately, they did not make further quantitative analysis or summary. Delayed postoperative elevation of cTnT suggests the persistent injury of cardiomyocytes after surgery, although there were no obvious clinical symptoms and the patients were discharged safely. This asymptomatic myocardial injury will also affect the long-term survival rate of patients undergoing OPCABG. When other factors in the multivariate Cox regression model were unchanged, the value and occurrence time of cTnT peak were included separately (Table 2). It was found that those two factors were independent risk factors of mid-term mortality. Most of studies only included the peak value when evaluating the risk factors of short- and long-term mortality, and ignored the peak occurrence time [5–7, 22]. Therefore, the peak value was considered as an independent factor of long-term mortality. Interestingly, when the time of peak occurrence was considered, the results changed. The multivariate Cox regression model including both peak value and peak occurrence time found only the peak occurrence time was an independent risk factor. This also indicates that the peak occurrence time is more predictive of mid-term mortality than the peak value. Delayed elevation of cTnT more greatly impacts mid-term prognosis than early elevation.

## Limitation

There are some limitations in this study. Firstly, the design single-centre retrospective study may cause selection bias. Secondly, the postoperative MIBs were also confected by preoperative myocardial injury (e.g. AMI), which was not considered in the article. Thirdly, the cohort size was small and the incidence of end point events in the follow-up period was also low, which may affect the statistical analysis of the data. Fourthly, the sensitivity and rang of cTnT need to be improved. This limitation will be made up for by the new-generation high-sensitivity cTnT applied in clinic [6, 9].

## Conclusions

The peak values and peak phases of postoperative MIBs are both risks factors for mid-term prognosis of OPCABG patients. Our data support the view that the peak phases are more powerful than the peak value in predicting mid-term prognosis. In addition, cTnT is superior over CK-MB in predicting mid-term prognosis.

## Data Availability

The datasets generated and analyzed during the current study are not publicly available due because it contains patient personal information but are available from the corresponding author on reasonable request.
